# Efficacy of Chlorine, QAC, and UV-C Light Against *Listeria monocytogenes* Biofilms on Food Contact Surfaces

**DOI:** 10.3390/foods15020347

**Published:** 2026-01-18

**Authors:** Manish Thapaliya, Jack N. Losso, Achyut Adhikari

**Affiliations:** School of Nutrition and Food Sciences, Louisiana State University, Baton Rouge, LA 70803, USA; mthapaliya@agcenter.lsu.edu (M.T.);

**Keywords:** *Listeria monocytogenes*, biofilm, food contact surfaces, UV-C

## Abstract

*Listeria monocytogenes* biofilms on surfaces that come into contact with food create ongoing challenges in produce-processing environments, highlighting the necessity for effective surface sanitation. This research examined the effectiveness of chlorine (200 ppm), quaternary ammonium compound (QAC, 400 ppm), and UV-C light (0.85 J/cm^2^) against *L. monocytogenes* biofilms developed on stainless steel, polyethylene terephthalate (PET), and silicone rubber materials frequently used in apple packing settings. Biofilms were cultivated using a mixture of LCDC and V7 strains in diluted apple juice and evaluated after 1 and 7 days of growth. The type of surface material and the age of the biofilm had a significant impact on the performance of the sanitizing agents (*p* < 0.05). Chlorine achieved a reduction of 2.84 ± 0.06 log CFU/coupon on 1-day-old biofilms on stainless steel, although its effectiveness dropped to 1.90 ± 0.07 log CFU/coupon on biofilms aged 7 days. Similar trends were noted for QAC (2.42 ± 0.05 to 1.73 ± 0.06 log CFU/coupon) and UV-C (2.71 ± 0.05 to 1.57 ± 0.08 log CFU/coupon) over time. PET and silicone rubber consistently exhibited lower log reductions than stainless steel for all treatments. The presence of organic matter from apple juice reduced the efficacy of sanitizers on all surfaces. These results emphasize the significant role of surface material, biofilm age, and organic load on sanitation effectiveness, offering practical recommendations for enhancing the control of *L. monocytogenes* in produce-processing facilities.

## 1. Introduction

*Listeria monocytogenes* is an environmental pathogen that poses a significant risk to food safety due to its versatility, ability to form biofilms on various surfaces, and association with high fatality rates during foodborne illness outbreaks [1]. This microorganism can thrive in various environments, including refrigerators and fresh and frozen produce surfaces, underscoring the importance of effective sanitation protocols in food processing facilities [2,3,4,5]. Nevertheless, the persistence of *L. monocytogenes* in processing facilities, especially through the development of biofilms on food contact surface materials such as silicone rubber, PET, and stainless steel, as noted by Buchanan, Gorris [6] and Bonaventura, Piccolomini [7], presents a significant obstacle to sanitation efforts.

Biofilms are protective layers that shield *L. monocytogenes* from environmental pressures and conventional sanitation measures. This makes them a potential source of ongoing food contamination [8,9]. Studies have shown that *L. monocytogenes* biofilms can resist sanitizers like chlorine and quaternary ammonium compounds (QACs), especially when the biofilm ages or contains organic matter. This phenomenon of resistance to antimicrobial interventions is well-documented [10,11]. To minimize cross-contamination and food surface contamination, the United States Food Safety Modernization Act (FSMA) has emphasized the importance of maintaining hygiene, especially for “covered” produce commonly consumed raw. Despite the recommendations, the long-term and cross-environmental efficacy of conventional chemical sanitizers against *L. monocytogenes* biofilms on various materials used in food processing environments, such as silicone rubber, PET, and stainless steel, remains uncertain.

In addition to conventional chemical sanitizers, ultraviolet (UV) radiation has been explored as a non-chemical strategy for inactivating microorganisms on food-contact surfaces. Sommers, Sites [12] and Koivunen and Heinonen-Tanski [13] have demonstrated the strong antibacterial properties of ultraviolet light, which effectively neutralize a wide range of microorganisms, even those protected by biofilms. Unlike traditional chemical sanitizers like chlorine and QACs, UV radiation does not leave any hazardous residues that could compromise the safety of food products. However, to optimize the application of these methods in industrial settings, a detailed examination of the dynamics of the biofilm response is necessary, including the response to the dose and duration of application of UV light and its impact on the survivability of the biofilm.

Despite the widespread use of chlorine and QACs and the growing interest in UV-C as a residue-free surface intervention, most previous studies have evaluated these treatments in isolation, on a single food-contact material, or under relatively clean laboratory conditions, often without realistic organic soiling [10,11,12,13]. Highlighting the comparison of these sanitizers across different materials and conditions can help industry professionals feel assured that the findings are relevant and comprehensive. As a result, there is limited information on how chlorine, QACs, and UV-C perform comparatively against *L. monocytogenes* biofilms of different ages (1 and 7 days) formed in the presence of organic matter on multiple food-contact surfaces. This gap is particularly critical for stainless steel, PET, and silicone rubber, which are widely used in produce handling and apple-packing environments and have been implicated in the persistence of *L. monocytogenes* on equipment and contact surfaces [6,14,15,16,17].

This research aimed to evaluate the efficacy of chlorine, QACs, and UV-C light against *Listeria monocytogenes* biofilms formed on stainless steel, PET, and silicone rubber surfaces under diluted apple juice conditions mimicking those encountered in apple-processing plants. Specifically, we compared the effectiveness of each sanitization strategy within and across these surfaces for 1-day- and 7-day-old biofilms to generate practical insights into the relative performance and limitations of these interventions for controlling *L. monocytogenes* biofilms in apple processing facility.

## 2. Materials and Methods

### 2.1. Inoculum Preparation

*Listeria monocytogenes* LCDC 81–861 (serogroup 4b, raw cabbage outbreak strain) and *L. monocytogenes* V7 (serogroup 1/2, a milk-associated outbreak strain) were used for this study. Each bacterial strain, stored at −80 °C, was thawed in a biosafety cabinet (Forma Scientific Inc., Marrieta, OH, USA), and 100 µL of the culture was transferred into 10 mL tryptricase soy broth (TSB)—0.6% yeast extract (TSBYE) and activated at 37 °C for 24 h followed by repeated activation for 48 h. A cocktail of two strains was prepared by mixing an equal volume of each activated strain. Each activated cocktail culture was pelletized by centrifuging at 2300× *g* for 15 min at 4 °C, rinsing it twice with 10 mL 1 × PBS Gu et al. [18], resuspending it in 5 mL 1 × PBS, and diluting the suspension until it showed the final concentration of McFarland 0.5 (approximately 10^8^ CFU/mL) in a McFarland Densitometer DEN1 (Grant Instruments, Royston, UK). The inoculum was adjusted to ~10^7^ CFU/mL (7 log CFU/mL) in 1:10 diluted apple juice, which was tested prior for any *Listeria* spp. contamination.

### 2.2. Food Contact Surface Preparation and Biofilm Formation

1.5 cm × 1.5 cm coupons of stainless steel (SS304, No. 4 polished, thickness: 0.03 in), PET (thickness: 0.03 in), and silicon rubber (thickness: 0.03 in) were used in this study. Each coupon was dipped overnight in a detergent solution, washed with tap water, and then immersed in 70% ethanol for 1 h to remove any grease. The coupons were then washed three times with sterile distilled water and kept in the biosafety cabinet (Forma et al., USA) to air dry. The coupons were sterilized by exposing them to UV-C light for 30 min (on each side) in the biosafety cabinet. The sterilized coupons were individually placed in each well of a 12-well tissue culture plate (Polystyrene, Costar, Corning, NY, USA). The well with coupons was filled with 2 mL of the inoculum in 1:10 diluted apple juice and incubated at 25 °C for 7 days.

### 2.3. Sanitize Treatment

Oasis Multi Quat Sanitizer (Ecolab, St Paul, MN, USA) was diluted with sterile water to prepare a 400 ppm QAC solution following the manufacturer’s recommendations. A 5% sodium hypochlorite solution was used for preparing a 200 ppm chlorine solution, while for the treatment with UV-C light, a UV lamp (6 W) was used as an emission source. These sanitizer concentrations and exposure times were selected to reflect commonly used, no-rinse food-contact surface sanitizing conditions in produce-processing and packing environments and to align with previous biofilm studies, which have typically used 100–200 ppm chlorine and 200–400 ppm QAC with 1–5 min contact times against *L. monocytogenes* on stainless steel and plastic food-contact materials [15,17,19,20]. The UV-C exposure (0.85 J/cm^2^ at 254 nm over 10 min, lamp positioned 5 inches above the surface) was chosen within the range reported to produce measurable reductions in *L. monocytogenes* and other foodborne pathogens on stainless steel and polymeric surfaces and is consistent with doses achievable using commercially available surface UV-C systems [21,22,23].

In this study, biofilms formed after 24 h incubation are referred to as 1-day (younger) biofilms, whereas those formed after 7 days incubation are referred to as 7-day (older/aged) biofilms. The coupons with 1-day- and 7-day-old mature biofilm were taken out from the 12-well plates and washed with 2 mL of 1 × PBS to remove the planktonic cells; the coupons were exposed to the two chemical sanitizers for 2 min and exposed for 10 min to the UV-C lamp, kept at a height of 5 inches above the surface, at an exposure dose of 0.85 J/cm^2^. The coupons were then immediately transferred into 10 mL of D/E neutralizing broth with 10 glass beads in a 50 mL falcon tube and vortexed for 2 min at maximum speed. The suspension was serially diluted and plated onto trypticase soy agar with 0.6% yeast extract (TSA-YE) and incubated for 48 h at 37°C and enumerated.

### 2.4. Statistical Analysis

The *L. monocytogenes* cell count obtained was converted to log CFU/coupon. The data were presented as mean value ± standard error of the mean averaged from three independent experiments (four replicates in each independent experiment). Analysis of variance (ANOVA) followed by a post hoc Tukey test were performed to evaluate the difference in the efficacy of the sanitizers employed across the different types of food-contact surfaces and biofilm ages at *p* < 0.05, using R studio version 4.2.1 (The R Foundation for Statistical Computing, Vienna, Austria).

## 3. Results

### 3.1. Biofilm Formation by L. monocytogenes

**Figure 1:**
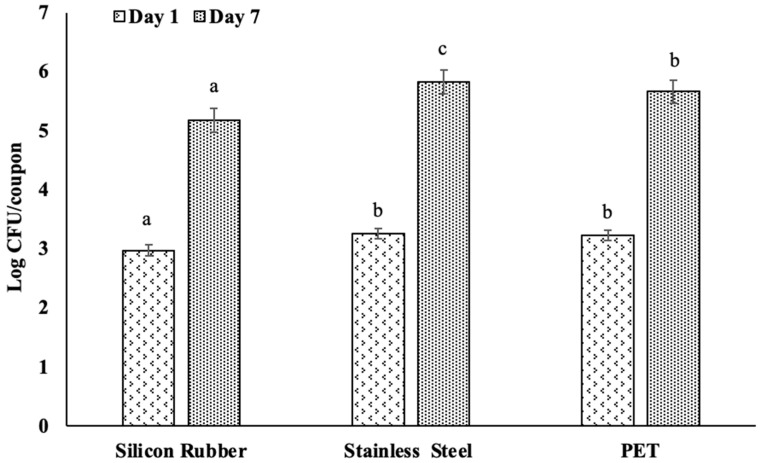
Comparative study of biofilm formation by *Listeria monocytogenes* on various food contact surfaces over time. The bar graph illustrates the log colony-forming units (CFU) per coupon on silicone rubber, stainless steel, and PET surfaces after incubating for 1 and 7 days in diluted apple juice. Lowercase letters indicate differences between surfaces within day of study. Surfaces with different letters had counts that were statistically significantly different (*p* < 0.05) for that day of study. Error bars represent standard errors.

The assessment of biofilm formation by *L. monocytogenes* on various food contact surfaces (silicone rubber, stainless steel, and PET) revealed distinct differences in bacterial accumulation between day 1 and day 7, Figure 1. These observations were quantified and analyzed under controlled experimental conditions at 25°C using diluted apple juice as the incubation medium.

After one day of incubation, we observed variations in attachment and biofilm formation. Silicon rubber showed the least biofilm formation, with an average of 2.97 log CFU/coupon. In contrast, stainless steel and PET surfaces exhibited slightly higher bacterial densities, with 3.25 and 3.23 log CFU/coupon, respectively, Figure 1. The statistical analysis indicated significant differences between the silicone rubber and the other two surfaces, with PET and stainless steel not differing significantly (*p* < 0.05).

Similarly, by day 7, all surfaces showed increased levels of *L. monocytogenes* counts. Stainless steel had the highest load, reaching an average of 5.82 log CFU/coupon, significantly higher (*p* < 0.05) than the 5.18 and 5.66 log CFU/coupon for silicone rubber and PET, respectively (Figure 1). This indicates that bacterial attachment and biofilm development by *L. monocytogenes* varies with the type of surface materials on which the biofilm develops, with stainless steel proving to be the best surface material for bacterial growth over time.

### 3.2. Efficacy of Chlorine

**Figure 2:**
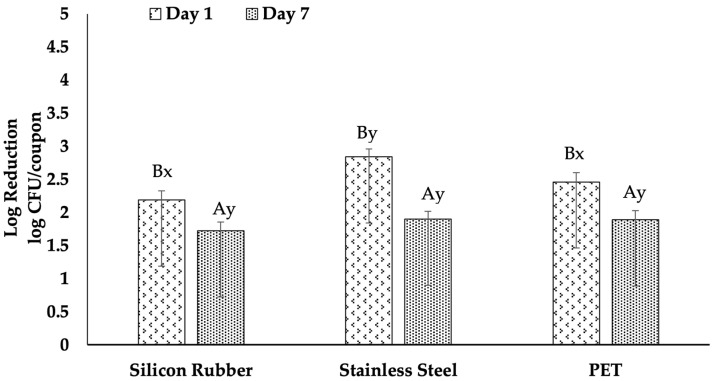
Efficacy of Chlorine treatment on *Listeria monocytogenes* biofilms across different surfaces. The graph displays the log reductions in colony-forming units (CFU) per coupon on silicone rubber, stainless steel, and PET surfaces treated with. Biofilms were analyzed at day 1 and day 7 post-inoculation. Uppercase letters (A, B) indicate significant differences in the efficacy of Chlorine treatment within each surface type over time, as determined by Tukey’s test (*p* < 0.05). Lowercase letters (x, y) denote significant differences in the efficacy of Chlorine treatment across different surfaces at the same biofilm age, also analyzed by Tukey’s test (*p* < 0.05). Error bars represent standard errors of the mean.

Analysis of the efficacy of chlorine treatment on various surfaces revealed notable reductions in *L. monocytogenes* biofilms Figure 2, albeit with varying degrees of success depending on the surface type and biofilm age. Specifically, a one-day-old biofilm on stainless steel exhibited a significant log reduction of 2.84 log CFU/coupon, which declined to 1.90 log CFU/coupon in the seven-day-old biofilm, indicating a statistically significant reduction in efficacy over time (*p* < 0.05). Similarly, chlorine’s efficacy decreased from 2.46 log reductions in one-day-old PET biofilms to 1.89 log reductions in seven-day-old biofilms (*p* < 0.05). In contrast, the log reductions on silicone rubber decreased from 2.31 log CFU/coupon to 2.02 log CFU/coupon, with statistically significant difference.

### 3.3. Efficacy of QACs

**Figure 3:**
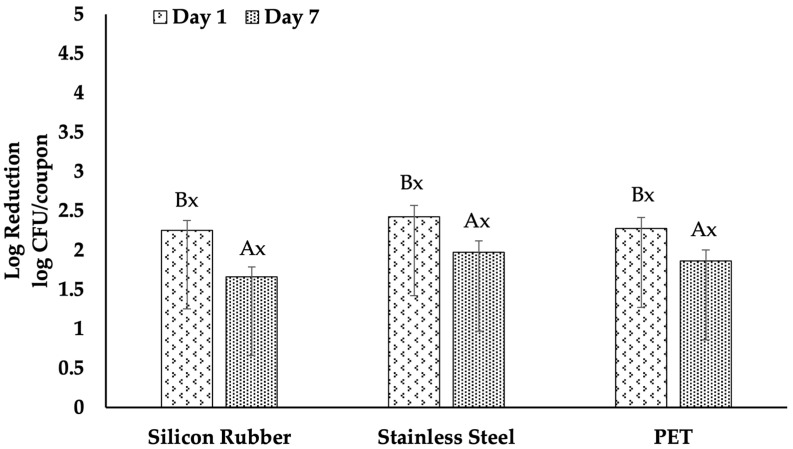
Efficacy of QAC treatment on *Listeria monocytogenes* biofilms across different surfaces. The graph displays the log reductions in colony-forming units (CFU) per coupon on silicone rubber, stainless steel, and PET surfaces treated with QAC. Biofilms were analyzed at day 1 and day 7 post-inoculation. Uppercase letters (A, B) indicate significant differences in the efficacy of QAC treatment within each surface type over time, as determined by Tukey’s test (*p* < 0.05). Lowercase letters (x) denote significant differences in the efficacy of QAC treatment across different surfaces at the same biofilm age, also analyzed by Tukey’s test (*p* < 0.05). Error bars represent standard errors of the mean.

All surfaces showed a significant reduction in the effectiveness of QAC treatments in reducing *L. monocytogenes* biofilms, with the efficacy decreasing with biofilm age Figure 3. The decrease in efficacy was more pronounced with increasing biofilm age. For instance, the log reduction in QAC on stainless steel was 2.42 for one-day-old biofilms, which dropped to 1.97 for seven-day-old biofilms, and the difference was statistically significant (*p* < 0.05). Similarly, the log reduction on PET was 2.27 for one-day-old biofilms and 1.86 for seven-day-old biofilms, with a significant difference (*p* < 0.05). The efficacy of QAC on silicone rubber decreased from 2.25 to 1.66 log reduction, which was also statistically significant (*p* < 0.05).

Moreover, the analysis demonstrated that the QAC treatment’s efficacy was consistent against one-day-old biofilms across all surfaces, as there were no statistically significant variations in the log reductions. Similarly, no statistically significant variations were observed in the seven-day-old biofilms across the three surfaces.

### 3.4. Efficacy of UV-C Light

**Figure 4:**
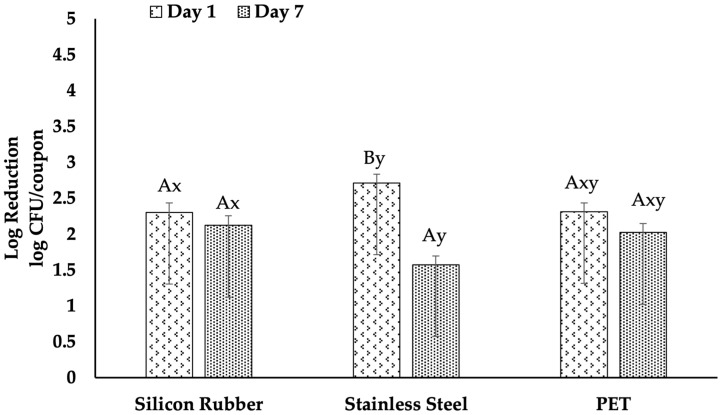
Efficacy of UV-C on *Listeria monocytogenes* biofilms across different surfaces. The graph displays the log reductions in colony-forming units (CFU) per coupon on silicone rubber, stainless steel, and PET surfaces treated with UV-C. Biofilms were analyzed at day 1 and day 7 post-inoculation. Upper case letters (A, B) indicate significant differences in the efficacy of UV-C treatment within each surface type over time, as determined by Tukey’s test (*p* < 0.05). Lowercase letters (x, y) denote significant differences in the efficacy of UV-C treatment across different surfaces at the same biofilm age, also analyzed by Tukey’s test (*p* < 0.05). Error bars represent standard errors of the mean.

Figure 4, illustrates the effectiveness of UV-C treatment in reducing the bacterial load from the biofilm formed on various surfaces of different ages. The results showed a statistically significant higher log reduction of 2.71 log CFU/coupon for one-day-old biofilms on stainless steel, compared to seven-day-old biofilms with a log reduction of 1.57 log CFU/coupon (*p* < 0.05). Similarly, a 2.31 log CFU/coupon reduction was observed for one-day-old biofilms formed on PET surfaces. In contrast, the log reduction for seven-day-old biofilms was 2.02 log CFU/coupon with no statistically significant decline (*p* > 0.05). In the case of silicone rubber, reductions ranged from 2.30 log CFU/coupon to 2.12 log CFU/coupon, with no statistically significant difference.

Furthermore, the results indicated that UV-C treatment was significantly more effective on one-day-old biofilms on stainless steel than PET and silicone rubber (*p* < 0.05). Additionally, statistically significant differences in the effectiveness of UV-C light were observed across the surfaces, which were evident for biofilms that were seven days old (*p* > 0.05), with the highest reduction in silicon rubber (2.12 log CFU/coupon) followed by PET (2.02 log CFU/coupon) and then in stainless steel (1.57 log CFU/coupon).

## 4. Discussion

Stainless steel is widely used in food processing and packaging equipment due to its exceptional corrosion resistance, which makes it an ideal material for facilities such as apple-packing plants [24]. However, rubber and PET are also significant materials as they are components of conveyor belts and brush beds, respectively. These materials come into direct contact with food, making them potential sites for microbial contamination. In particular, *L. monocytogenes*, a pathogen known to form biofilms, has been found to thrive on these surfaces, complicating sanitation efforts despite daily cleaning with chemical sanitizers [25,26].

*Listeria monocytogenes* could attach and form biofilms on all the surfaces employed in the study. However, a significant variation was observed in the biofilm-forming ability of *L. monocytogenes* among the surfaces, with stainless steel being a better biofilm-supporting surface than PET and silicon rubber. These findings are consistent with those reported by Bonsaglia, Silva [27], who observed that hydrophilic materials like glass and stainless steel supported higher biofilm formation than hydrophobic materials like polystyrene. Their study highlighted that the ability of *L. monocytogenes* to form biofilms is influenced by the surface properties, with hydrophilic surfaces promoting faster biofilm development regardless of the incubation temperature. Similarly, Blackman and Frank [28] reported significant biofilm accumulation on stainless steel and other materials like Teflon™, indicating that these surfaces’ physical and chemical properties facilitate bacterial adherence and growth.

Surface hydrophobicity and roughness are crucial factors impacting biofilm formation in different materials. Hua, Younce [16] demonstrated that surfaces with higher hydrophobicity, such as rubber, exhibited greater resistance to biofilm formation, while hydrophilic surfaces like stainless steel were more prone to bacterial colonization. This aligns with our observations, where stainless steel showed the highest biofilm accumulation over time.

### 4.1. Comparative Efficacy of Chlorine, QAC, and UV-C on Different Surfaces

Our research found that treating surfaces with 200 ppm of chlorine for 2 min resulted in a significant decrease in *L. monocytogenes* biofilms on the different surfaces. However, the effectiveness varied based on the surface material and the age of the biofilm. For 1-day-old biofilms, the log reduction was significant across stainless steel (2.71 log CFU/coupon), PET (2.31 log CFU/coupon), and silicone rubber (1.89 log CFU/coupon). However, for 7-day-old biofilms, the reduction was less effective, with the load decreasing to 1.57 log CFU/coupon on stainless steel, 1.89 log CFU/coupon on PET, and 2.02 log CFU/coupon on silicone rubber. These findings align with [17], who also observed reduced efficacy of chlorine at similar concentrations on older biofilms across different surfaces. Additionally, Korany, Hua [19] demonstrated that chlorine at 200 ppm reduced *L. monocytogenes* biofilms by 2.0–3.1 log CFU/coupon on polystyrene, showing a similar pattern of reduced effectiveness as biofilms aged. This reduced effectiveness over time highlights the challenge of relying solely on chlorine to combat mature biofilms effectively.

The results of our study showed that for QAC treatment, there was a 2.61 log CFU/coupon reduction for 1-day-old biofilms on stainless steel, which decreased to 1.97 log CFU/coupon for 7-day-old biofilms. PET and silicone rubber surfaces exhibited reductions of 2.31 and 2.27 logs for 1-day-old biofilms, respectively, with a similar decline in efficacy observed for 7-day-old biofilms. These findings are consistent with those obtained by Pan, Breidt, Jr. [29] who observed that QAC effectiveness was lower on older biofilms, highlighting the persistent nature of *L. monocytogenes* in biofilm. Furthermore, Hua, Korany [15] noted that increasing QAC concentrations or extending contact times improved efficacy, yet the effectiveness against aged biofilms remained limited. This supports our findings that a simple QAC treatment may not be sufficient for older biofilms.

Our findings showed that UV-C light was most effective in reducing biofilms on stainless steel that were 1 day old, achieving a reduction of 2.71 log CFU/coupon. However, this effectiveness decreased as the biofilm aged to 7 days, resulting in only a 1.57 log CFU/coupon reduction. The efficacy of UV-C on PET and silicone rubber was lower than on stainless steel on a one-day-old biofilm but better on PET and silicone rubber than on stainless steel on a seven-day-old biofilm. This trend aligns with the results of Harada and Nascimento [21], who found that UV-C was more effective on polypropylene, a material similar in hydrophobicity to PET, compared to stainless steel. The differences in UV-C effectiveness between these studies could be attributed to surface properties such as roughness and the presence of micro-scratches, which can protect microbial colonies from direct UV-C exposure, as noted by Bintsis, Litopoulou-Tzanetaki [30].

### 4.2. Effect of Surface Properties on Chlorine, QAC, and UV-C

While surface roughness and hydrophobicity were not assessed directly in this study, they probably played a role in the variations in sanitizer effectiveness that we observed. Our understanding of these influences aligns with earlier findings regarding the properties of food-contact materials like stainless steel, PET, and silicone rubber. Surface properties significantly influenced the effectiveness of chlorine treatments. Stainless steel, which is smooth and hydrophilic, allowed for better penetration and action of chlorine, leading to higher log reductions than silicon rubber especially in one-day-old biofilms. This is because silicon rubber has a rougher texture and more hydrophobic properties. Hua and Zhu [17] discovered that worn or abraded surfaces on stainless steel reduced the efficacy of chlorine, indicating that maintaining surface integrity is crucial for effective sanitization. Similarly, Hua, Korany [15] and Park and Kang [31] observed that chlorine was more effective on smoother surfaces, which supports our findings that surface roughness and defects significantly hinder the effectiveness of chlorine.

The impact of surface properties on QAC treatments was evident in our study. We observed that QAC was less effective on silicon rubber than stainless steel, likely due to silicon rubber’s hydrophobic and rough nature. Krysinski, Brown [32] reported that surfaces like polyurethane and polyester, which are more prone to scratches and abrasions, protected *L. monocytogenes* biofilms against QAC, reducing its efficacy. Our observations also support the idea that surface type and condition significantly influence the outcome of QAC sanitization.

The varying effectiveness of UV-C on different materials is linked to their physical characteristics. Stainless steel, despite its smoother surface, often contains micro-scratches that create a shadowing effect, reducing UV-C penetration [33]. On the other hand, materials like PET, which are generally smoother and less prone to scratching, may allow more consistent UV-C exposure. Surface hydrophobicity and roughness were crucial in determining the bactericidal efficacy of UV-C LEDs, leading to varying degrees of microbial inactivation [22]. However, these differences were not statistically significant in our study.

### 4.3. Effect of Age of Biofilm on the Efficacy of Chlorine, QAC, and UV-C

The age of the biofilm was identified as a critical factor in determining chlorine’s effectiveness. Our research indicated that chlorine was notably less successful in combating 7-day-old biofilms than 1-day-old biofilms on all surfaces. This result is consistent with the findings of Hua and Zhu [17], who showed that older biofilms were more resistant to chlorine, requiring higher concentrations or longer exposure times to achieve similar reductions as younger biofilms. Similarly, Pan, Breidt, Jr. [29] highlighted the heightened resistance of aged biofilms to chlorine, emphasizing the necessity for more aggressive or combined treatments to control biofilms effectively. Mechanistically, prolonged incubation allows *L. monocytogenes* biofilms to form a thicker and more interconnected extracellular polymeric substance (EPS) matrix, leading to a more complex three-dimensional structure. This increased thickness hinders the penetration of hypochlorous acid into the deeper layers and heightens the local chlorine demand within the matrix [34,35,36]. Additionally, cells residing in these older, nutrient-scarce areas tend to grow at a slower pace and exhibit stress-adapted characteristics, making them less vulnerable to oxidizing agents that primarily target metabolically active cells [10,15,37]. This protective effect of the EPS and the resulting physiological variability help clarify why a chlorine treatment of 200 ppm for 2 min, which yielded notable reductions in 1-day biofilms, was less effective in producing significant log reductions in 7-day biofilms in our research.

In our study, we also observed a decrease in the efficacy of QAC as biofilm age increased. The resistance of 7-day-old biofilms to QAC was significantly higher compared to 1-day-old biofilms, resulting in significant reductions in log CFU across all tested surfaces. Similar findings were reported by Korany, Hua [19] and Pan, Breidt, Jr. [29], indicating that older biofilms were more resistant to QAC. Cationic surfactants, specifically quaternary ammonium compounds (QACs), primarily target and disrupt bacterial membranes. However, in older biofilms, a significant portion of QACs may be sequestered or neutralized by negatively charged extracellular polymeric substances (EPS) and conditioning layers before they can reach the bacterial cell surfaces [35,36]. Additionally, aged biofilms of *L. monocytogenes* are more likely to harbor persistent subpopulations and cells that express resistance mechanisms, such as the qacH, bcrABC, or emrE efflux systems. These mechanisms are associated with diminished susceptibility to benzalkonium chloride and similar QACs [37,38,39,40]. Consequently, the combined effect of matrix sequestration, efflux-mediated resistance, and the presence of slow-growing or dormant cells explain the lower efficacy of QACs observed in 7-day biofilms compared to those observed in 1-day biofilms.

The age of the biofilm has a significant impact on the effectiveness of UV-C treatments. Older biofilms are more resistant, possibly because they are denser and more complex, which can make it harder for UV-C light to penetrate. This finding is consistent with von Hertwig, Prestes [41], who observed that UV-C radiation’s effectiveness varied depending on the age of the biofilm, with decreased efficacy on older biofilms. As biofilms develop thicker layers, the extracellular polymeric substances (EPS) and cell clusters on the surface can absorb and scatter ultraviolet (UV) light, resulting in a shadowing effect that shields the deeper cells from direct exposure to radiation [21,22]. Additionally, older biofilms provide cells with more time to initiate DNA repair mechanisms and general stress-response pathways, allowing for better recovery of sublethally damaged cells from UV-induced injuries like pyrimidine dimers [34,37]. The increased resistance of older biofilms may also be due to the accumulation of extracellular polymeric substances (EPS), which can absorb or scatter UV-C light, reducing its impact on the bacterial cells within the biofilm [42]. These structural and physiological alterations observed in aged biofilms align with the slight logarithmic reductions achieved with a single dose of UV-C, especially in the 7-day biofilms developed with apple juice.

### 4.4. Role of Organic Matter

In our research, using apple juice as the medium for biofilm formation may have significantly reduced the effectiveness of the treatments. The organic matter in the apple juice could have formed a physical barrier around the biofilm, making it difficult for chlorine and QAC to reach and inactivate the bacterial cells. This aligns with the findings of Hua, Korany [15], which showed that surfaces soiled with apple juice or milk significantly impacted the antimicrobial effectiveness of QAC and chlorine against *L. monocytogenes* biofilms. Organic residues alter the physical and chemical characteristics of surfaces that come into contact with food and significantly impact the effectiveness of sanitizers. Hua et al. [15] noted that organic contaminants, irrespective of their origin, reduced the effectiveness of all sanitizers used in the study on various materials. This observation reinforces our finding that even a single type of organic residue, like apple juice, can considerably hinder sanitizer effectiveness in conditions relevant to processing.

In addition to Hua et al. [15], several other studies have indicated that residues high in protein and fat on stainless steel and other surfaces diminish the effectiveness of various sanitizers, such as chlorine dioxide, hydrogen peroxide, acidic electrolyzed water, sodium hypochlorite, quaternary ammonium compounds (QACs), chlorine, and peroxyacetic acid [8,12,39,43,44]. These residues not only attract bacterial cells and create an adhesive conditioning layer but also lower the water contact angle and modify the surface’s hydrophobicity. This alteration can influence the spreading of liquids and the interaction of sanitizers with the surface [31,45]. When combined with the exopolysaccharide matrix of biofilms, this conditioning layer hinders the ability of sanitizers to penetrate deeper layers and effectively eliminate embedded cells [46].

In addition to simply preventing access, organic matter alters the interactions of microorganisms with physical and chemical treatments. Bernbom et al. [47] found that *L. monocytogenes* that adhered to smoked salmon extract endured UV-C treatment much better than cells attached to tryptic soy broth, regardless of the presence of 5% NaCl. They suggested that food residues offer extra protection for surface-attached cells due to both a more substantial extracellular matrix and the ability of organic materials to absorb and lessen UV-C radiation, thereby limiting its penetration into the surrounding microenvironments of the cells [47,48]. Similar findings have emerged from disinfection studies conducted on hospital and industrial surfaces, showing that organic loads significantly diminish the effectiveness of UV-C and chemical disinfectants. Pre-cleaning to eliminate soil is consistently recommended as a vital step for ensuring reliable microbial inactivation [48,49]. In our scenario, residues from apple juice on the coupons likely served a similar purpose, conditioning the surface and embedding the biofilm within an organic matrix that obstructs the diffusion of sanitizers and the penetration of light.

Specifically, regarding UV-C, organic matter affects not only surface shielding but also the optical characteristics of the liquid film covering biofilms. Sharma et al. [50] demonstrated how the absorbance, scattering, and thickness of the film over stainless steel coupons dictate UV-C intensity gradients within droplets, revealing that photons are absorbed mainly in the upper layers of the film, while lower regions receive significantly reduced doses. Similarly, Ramos et al. [51] reviewed how food composition, turbidity, and suspended solids greatly influence UV-C D-values in various matrices by absorbing or scattering radiation before it reaches microbial cells. For our biofilms grown in apple juice, this indicates that even when the intended surface dose remained consistent, the effective dose reaching cells embedded within the EPS-organic matrix was likely much less than the applied 0.85 J/cm^2^, which helps clarify the modest log reductions seen for UV-C in the presence of organic matter.

Overall, these insights emphasize that organic contamination and biofilm EPS work together to create barriers to both diffusion and optical penetration. Residual juice solids, proteins, and other large molecules condition the surface, trap cells, and absorb or scatter UV-C, while simultaneously hindering the diffusion of chlorine and QACs and neutralizing reactive species [15,47,48]. Practically speaking, this indicates that effective management of L. monocytogenes biofilms in apple-processing environments will necessitate thorough pre-cleaning to remove organic deposits and disrupt the matrix before the application of sanitizers or UV-C, rather than depending on a single-step disinfection approach on heavily contaminated surfaces.

### 4.5. Limitation and Future Research

This study has several significant limitations. We utilized a two-strain, mono-species *L. monocytogenes* cocktail, which was cultivated as static biofilms for 1 and 7 days in diluted apple juice on new stainless steel, PET, and silicone rubber coupons. However, in actual commercial apple-packing settings, biofilms are typically mixed-species, older than 7 days, and subjected to factors such as flow, temperature changes, and worn or damaged surfaces, all of which are known to enhance sanitizer tolerance [15,19,29,37]. Additionally, our study only assessed one regulatory-compliant concentration and contact time for chlorine (200 ppm, 2 min) and QAC (400 ppm, 2 min), alongside a single UV-C fluence (0.85 J/cm^2^) without any prior cleaning steps. We did not directly measure surface roughness, hydrophobicity, or biofilm architecture; therefore, our understanding of surface-dependent effects relies on previously reported properties of these materials rather than direct physical characterization of the coupons used [16,17,27,28].

For future research, it would be beneficial to (i) include mixed-species biofilms and environmental Listeria isolates collected from produce facilities; (ii) explore a wider range of sanitizer concentrations, contact times, and UV-C doses as well as sequential cleaning-sanitizing protocols and combined interventions like using sanitizer with UV-C or mild heat [21,23]; and (iii) specifically analyze surface wear, roughness, and hydrophobicity of both new and aged equipment materials. Correlating this data to product contamination and consumer risk through quantitative microbial risk assessment for apple and caramel-apple scenarios would aid in translating 3-log reductions at the surface into more significant estimates of real-world risk reduction.

## 5. Conclusions

This study indicated that the effectiveness of chlorine (200 ppm, 2 min), QAC (400 ppm, 2 min), and UV-C (0.85 J/cm^2^) in combating *L. monocytogenes* biofilms was significantly influenced by the type of surface and the age of the biofilm, particularly in the presence of organic matter from apple juice. Stainless steel surfaces exhibited the highest biofilm formation and proved to be the most difficult to clean, whereas silicone rubber generally accumulated less biofilm. Targeting high-risk surfaces, such as stainless steel, is crucial, as biofilms aged 7 days were considerably more resistant than those just 1 day old, with none of the treatments achieving a ≥3-log reduction under contaminated conditions.

Practically, these results suggest that relying solely on a single chemical sanitizer or UV-C treatment at specified concentrations is insufficient once Listeria biofilms have matured and are entrenched in organic residues. In environments like apple processing and fresh produce, control measures should emphasize: (i) regular and thorough cleaning to eliminate juice residues before using sanitizers; (ii) prompt action to address biofilms before they develop into more resilient stages; and (iii) diligent monitoring and upkeep of high-risk surfaces like stainless steel equipment. Future research into combined or sequential treatment methods (such as cleaning followed by sanitizer or sanitizer coupled with UV-C or heat) on degraded or worn surfaces will be critical for translating these laboratory findings into effective sanitation practices in commercial settings.

## Data Availability

The original contributions presented in the study are included in the article, further inquiries can be directed to the corresponding author.

## Abbreviations

The following abbreviations are used in this manuscript:

ANOVA	Analysis of variance
CFU	Colony-forming unit
EPS	Extracellular polymeric substances
FSMA	Food Safety Modernization Act
PBS	Phosphate-buffered saline
PET	Polyethylene terephthalate
QACs	Quaternary ammonium compounds
TSB	Trypticase soy broth
TSB-YE	Trypticase soy broth–yeast extract
TSA	Trypticase soy agar
TSA-YE	Trypticase soy agar–yeast extract
UV-C	Ultraviolet-C
